# Cardiogenic oscillation in pediatric patients after cardiac surgery

**DOI:** 10.1186/cc13371

**Published:** 2014-03-17

**Authors:** H Imanaka, N Okuda, T Itagaki, M Onodera, M Nishimura

**Affiliations:** 1 Tokushima University Hospital, Tokushima, Japan

## Introduction

Cardiogenic oscillation is the fluctuation in flow tracing in mechanically ventilated patients. Large cardiogenic oscillation may cause autotriggering in adult patients after cardiac surgery [1] and inaccurate volume monitoring [2]. However, it is unknown how cardiogenic oscillation is problematic in pediatric patients. Therefore, we prospectively surveyed cardiogenic oscillation in pediatric patients after cardiac surgery.

## Methods

We enrolled 17 pediatric patients who underwent cardiac surgery using cardiopulmonary bypass. They were mechanically ventilated with pressure-controlled ventilation. We measured the amplitude in cardiogenic oscillation and compared them between their admission to the ICU and before extubation. We performed statistical analysis with the *t *test and considered *P *< 0.05 significant.

## Results

**Figure 1:**
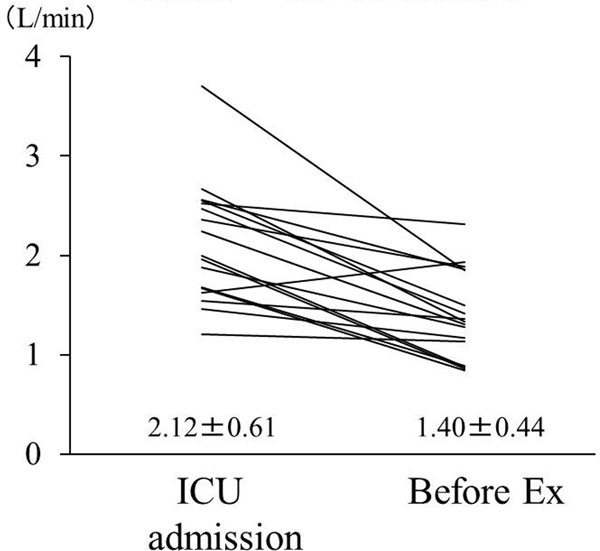
**Cardiogenic oscillation**.

Cardiogenic oscillation was 2.1 ± 0.6 l/minute just after the surgery (Figure 1). Autotriggering occurred in seven of 17 patients when triggering sensitivity was set at 1 l/minute. Before the extubation, cardiogenic oscillation significantly decreased to 1.4 ± 0.4 l/minute when autotriggering disappeared. Intensive care including adjustment of inotropes and intravascular volume might have contributed to the decrease in cardiogenic oscillation.

## Conclusion

In pediatric patients after cardiac surgery, cardiogenic oscillation was initially large but was decreasing at the extubation.
